# Clinical Lecture upon Surgical Cases in the Buffalo Hospital of the Sisters of Charity

**Published:** 1871-12

**Authors:** J. F. Miner


ART. III.—*Clinical Lecture upon Surgical Cases in the Buffalo Hospital of the Sisters of Charity*, by Prof. J. F. MINER.

Reported by LOUIS BRECHT, member of the class.

CASE VIII.—STRABISMUS.—J. Brown, aged 21, has come to our clinic for the purpose of having his right eye operated upon, for convergent Strabismus. You will see that both his eyes seem to be turned inwards, but closer examination in different positions will show you that the right eye is the one principally affected. Strabismus is a very common condition of deformity and greatly impairs good appearance. It may be hereditary, may be caused by injury or disease of the brain or nerves, and may be in various ways acquired. The deformity should be corrected as early as possible, for if the brain neglects to take cognizance of the image which is formed upon the retina for a great length of time, vision will be lost, the eye gradually becoming incapable of vision from long disuse. When one eye is affected, the other will often, in some degree accommodate itself to the same axis and appear as if also affected, this deviation is called concometant squint, and will be fully explained hereafter; both eyes are sometimes similarly affected. The operation in the present case consists in dividing the tendon of the internal rectus muscle. For this purpose I make a small transverse incision through the conjunctiva through which I pass the hook, and taking up the tendon of the muscle divide it near its insertion, passing the scissors through the small opening in the conjunctiva. I also divide if necessary the subconjunctival tissue which seems to prevent in some degree the return of the eye to its normal position. This is not what most authors call subconjunctival operation, which is done by passing a hook shaped knife under the conjunctiva and dividing the tendon. Formerly large incisions were made through the conjunctiva, in this way exposing the sclerotic coat, and allowing the caruncle to retract thus aiding to, rather than diminishing the deformity; lately, however the operation is greatly modified, as you have seen this morning. A well conducted operation for Strabismus, simple as it may appear to you, requires skill and care on the part of the surgeon, for it is vastly more apt to cause deformity greater than Strabismus itself if discrimination is not exercised. You observe that the eye most turned is now straight, while the other eye shows slight convergance; in time both may assume positions in harmony with each other.

Case IX.—Strabismus.—G. Miller, aged 22 years, comes before you for the same operation. The deformity is the same and we will operate in the same manner.

Case X.—Strabismus.—The third patient, Mary G., a little girl 8 years of age, also requires operation for Strabismus. From her history I gather that she received a fall some years ago from which time the deformity commenced. We will operate as before, with the exception of placing the little patient under chloroform while the former two patients suffered the pain of the operation without it. The patient should be entirely under its influence, for if only partially so greater restlessness is present than if none had been given.

CASE XI.—NON-UNION OF FRACTURE.—Thomas Lincoln is 45 years of age,has an ununited fracture of the right humerus. He received a compound fracture near the middle third of the shaft of the bone in May last, while working on a gravel train. He entered the hospital immediately after receiving the injury and was treated with all the advantages available, yet no union could be effected. You will observe that a second joint exists above the elbow at the seat of fracture, which is caused by a ligamentous connection. Non-union of a fracture is not uncommon; it may occur in any bone of the body, more frequently, however, in the humerus and thigh. The causes of non-union may be constitutional or local. Among the former may be enumerated inherited and chronic diseases, and impaired nutrition. But there is no ground to apply these causes in the present case, for the patient is a healthy and well nourished man. Among the latter causes may be mentioned undue motion of the parts and separation of the fragments by intervention of muscle or other substance. Patients are very apt to blame the attending surgeon for such results, and to a surgeon in the country, where he is surrounded by jealous neighbors and doctors, such results would be very vexatious. Yet I do not wish to entirely exhonerate surgeons from such results, for bad dressing sometimes may be a cause; but oftener, in the majority of cases, the patients own carelessness, in undue motion, restlesness and uneasiness are the most active causes. In many instances, no adequate cause can be assigned. The one before you is of this nature.

Various methods of treatment have been recommended. Scraping the ends of the bones was employed at a very early period. This method was later improved by cutting off the ends and tying the fragments together with metallic wire. Dr. Brainard,of Chicago, employed a metallic perforator, in the shape of an awl, by means of which he bored through and destroyed the ligament. Resections of the ends of the shaft have been practiced, and I have myself pracitced it with some success. If bony union never takes place, the arm may still be made useful by suitably adjusted splints placed so as to envelope the arm. A seton passed through the cartilaginous band between the fragments was, it is said, first suggested by Dr. Winslow in 1787, but first successfully employed by Dr. Physick in 1802. The procedure is very simple and should, perhaps, be tried before any severer method. You observe that the seton, as now introduced, is passed with much greater difficulty than if passed through the fleshy part of the arm. It is designed to cause inflammation and the deposit of bony material. The arm will now be placed in immovable pasteboard dressings, and time afforded for bony union. Should this treatment fail, any of the other methods may be employed.

CASE XII.—SYPHILIS.—The patient before you entered the hospitable last Spring soon after the close of the lecture term, with immense enlargement of the testicles. One was suppurating and would probably weigh two pounds. He denied ever having had any private disease, and nothing of his history could be gained. We removed the testicle most diseased and allowed the other to remain; it has lessened in size, but is now the site of suppurative disease, and might as well have been removed. Aside from this history, as this patient is presented, it is an illustrative case of tertiary Syphilis. Now, gentlemen, what has produced this suppurative disease which has been going on in the testicles, and which has recently appeared on other parts, especially upon the face? Patients consult you and say boldly they have "pox," they also ask your advice and firmly deny having had any such disease; the one is worth as much to you as the other, so far as enabling you to arrive at any conclusion. Those who say they have had "pox" are often, very often mistaken, and those who say they have not, may intend to deceive. You are always then to go back in the history a little and ascertain if posible from what they have previously suffered.

The primiary lesion of Syphilis is essential to the secondary and tertiary symptoms; but the primary lesion may have been in the urethra or elsewhere out of sight, and is then called *concealed.* It may have been so slight and have produced so little irritation, as not to have attracted attention: but if there is Syphilis there has been a point of infection—*the primary lesion of Syphilis.* Following this there are general symptoms—eruptions upon the skin, sore throat, falling of the hair, rheumatic pains, general fever and emaciation—some, or all of these symptoms appear, generally within the first year after the infection, this constitutes what is called secondry Syphilis.

Later the disease affects the internal organs and bones. These later symptoms are grouped into what is called *tertiary Syphilis.* I do not propose this morning to minutely describe Syphilis, but to call your attention to the order of its "stages" so-called, and to explain the nature of the syphilitic node you observe upon the patient before you. *Nodes* appear chiefly upon tibia, ulna and skull, but here we find it upon the malar bone. It consists of a specific inflamamation affecting the periosteum and sometimes bone giving rise to effusions of lymph, serum or a peculiar gummy deposit. These deposits undergo various changes, which mainly determine the varieties of the disease described by authors. Before you is a *suppurative node,* and from its extent, long standing and general appearance, it is feared that the bone itself is becoming involved in caries.

TREATMENT.—The treatment of this affection is the treatment of tertiary Syphilis. I have repeatedly told you that mercury is the only specific for Syphilis, and that though the disease will in some instances terminate favorably if left to nature alone, yet mercury does exercise an almost specific influence. If medicine has curative influence uponany disease,it is mercury in secondarySyphilis and when I am forced to believe otherwise, I shall also conclude that medicine is useless, and that all our observations have but blinded our eyes and led us more deeply into error. I am not insensible of the great worth and high standing of those who deny its curative power, and affirm that Syphilis can be better treated without it, but I assure you that if you prescribe it in suitable manner and time, you will be more and more impressed with its favorable effects. Blue mass is, according to my observation, the best preparation of the drug, though in all forms it is about equally efficacious. When you have obtained the effect of mercury upon the system, as shown by tenderness of the teeth and gums, you have in almost every instance obtained marked abatement or total disappearance of all the symptoms of secondary Syphilis. In the later stages and after full trial of mercury, you may suppliment it with iodide of potassium, a drug which has been more indiscriminately employed than almost any other. It is of signal service inthe management of all the later or tertiary manifestations of the disease, and as the case before you is of that nature we shall prescribe it, as at this stage, more suited to the case than any other remedy.

